# Efficacy and safety of add-on treatment with finerenone in patients with diabetic kidney disease already treated with SGLT-2 inhibitors 

**DOI:** 10.5414/CNP104S16

**Published:** 2025-11-28

**Authors:** Manja Antonič, Anton Adamlje, Boštjan Leskovar, Denis Fornazarič

**Affiliations:** 1 Department of Dialysis, General Hospital Trbovlje, Trbovlje, and; 2 Department of Nephrology, University Medical Center Ljubljana, Ljubljana, Slovenia

**Keywords:** chronic kidney disease, diabetes, renoprotection, SGLT-2 inhibitors, finerenone

## Abstract

Introduction: This study evaluated the effectiveness and safety of an add-on treatment with finerenone as combination therapy in patients with diabetic kidney disease (DKD), an area where real-world data is limited. Materials and methods: We retrospectively evaluated patients with DKD treated with sodium-glucose cotransporter 2 (SGLT-2) inhibitors and add-on finerenone, to assess the effectiveness and safety of the combination therapy (data collected between June 2021 and October 2024). Outcomes included changes in urinary albumin-to-creatinine ratio (UACR), protein-to-creatinine ratio (UPCR), estimated glomerular filtration rate (eGFR), and serum potassium before and after finerenone initiation. Results: Among 23 patients (mean age 72 ± 7; 17 male) on SGLT-2 inhibitors, 21 (91%) also received renin-angiotensin system (RAS) inhibitors and 9 (39%) glucagon-like peptide-1 (GLP-1) receptor agonists. Addition of finerenone significantly reduced UPCR from 0.52 (0.18 – 1.35) to 0.41 (0.16 – 1.78) g/g (p = 0.046), a median decrease of 35% (IQR –53 to –8). UACR showed a reduction trend from 285 (36 – 1,020) to 266 (57 – 1,006) mg/g (p = 0.15), with a median decrease of 36% (IQR –65 to +14). Kidney function remained stable with a small non-significant decline in eGFR (45 ± 22 to 44 ± 21 mL/min/1.73m^2^; –4% ± 13%; p = 0.13). Serum potassium increased slightly but significantly (4.3 ± 0.5 to 4.5 ± 0.4 mmol/L; p = 0.045), with 1 mild hyperkalemia case (5.6 mmol/L) and no treatment discontinuations. Conclusion: In this real-world cohort, the renoprotective combination therapy with added finerenone was associated with a further reduction in albuminuria and proteinuria. The treatment was well tolerated with a minimal increase in potassium levels and generally stable renal function.

## Introduction 

Although renin-angiotensin system (RAS) inhibitors and sodium-glucose cotransporter 2 (SGLT-2) inhibitors reduce proteinuria and offer renoprotection, patients with diabetic kidney disease (DKD) remain at residual risk for chronic kidney disease (CKD) progression and cardiovascular complications. Newer therapies, such as the selective non-steroidal mineralocorticoid receptor antagonist (MRA) finerenone, offer additional benefits through complementary mechanisms [1]. 

While RAS and SGLT-2 inhibitors primarily lower intraglomerular pressure and albuminuria [1, 2, 3, 4], finerenone uniquely targets inflammation and fibrosis, contributing to further slowing of kidney function decline and cardiovascular risk reduction [5, 6]. Combining these agents may therefore optimize renoprotection. Glucagon-like peptide-1 (GLP-1) receptor agonists also exert antifibrotic and antioxidative effects, improving cardiorenal outcomes [1, 7]. 

Updated CKD guidelines for patients with type 2 diabetes (T2D) support combination pharmacotherapy [8]. Though randomized trials have shown the individual benefits of SGLT-2 inhibitors and finerenone, evidence on their combined use is limited [2, 4, 5, 6]. In FIDELIO-DKD, only 4.4% of patients on finerenone were also treated with an SGLT-2 inhibitor [5], but post-hoc and subgroup analyses suggest synergistic effects when used together [9, 10, 11, 12]. Observational studies support these findings in both diabetic and non-diabetic CKD populations [13, 14]. 

In clinical practice, finerenone is increasingly added to regimens with RAS and SGLT-2 inhibitors, sometimes alongside GLP-1 receptor agonists, reflecting the “4 pillars” approach in CKD and T2D management [8, 15]. 

In our retrospective study, we assessed the real-world effectiveness and safety of a regimen involving SGLT-2 inhibitors with subsequent addition of finerenone on top of RAS inhibitors and GLP-1 receptor agonists, in an outpatient setting. 

## Materials and methods 

We conducted a retrospective study of 23 patients with CKD and T2D, treated at outpatient nephrology clinics at two centers in Slovenia: 18 patients at General Hospital Trbovlje and 5 patients at University Medical Center Ljubljana. These patients were managed individually by four different nephrologists. Data covered the observation period from June 2021 to October 2024. 

### Patient selection and inclusion criteria 

The cohort included all known patients with DKD under each nephrologist’s care who were all treated with an SGLT-2 inhibitor followed by the addition of finerenone and who had at least one follow-up visit after starting the combination therapy with finerenone. 

All patients initially received an SGLT-2 inhibitor, either before or at their first nephrology visit. Finerenone was then added sequentially during follow-up at the outpatient nephrology clinic. Most patients were already on RAS inhibitors before starting SGLT-2 therapy and continued both treatments after finerenone was introduced. Additionally, some patients were treated with a GLP-1 receptor agonist at the time of data collection. The diagnosis of diabetic kidney disease was made clinically, based on the nephrologist’s assessment. The intervals between the laboratory time points varied depending on each nephrologist’s approach to patient follow-up. 

### Exclusion criteria 

Patients who received only one of the two medications (either an SGLT-2 inhibitor or finerenone) or initiated both therapies simultaneously were excluded from the study. 

### Data collection on effectiveness and safety 

The effectiveness of treatment was assessed using albuminuria (urinary albumin-to-creatinine ratio (UACR); mg/g) and proteinuria (urinary protein-to-creatinine ratio (UPCR); g/g), while safety was evaluated by monitoring estimated glomerular filtration rate (eGFR), serum potassium, and reported side effects, at 2 time points: before starting finerenone (LAB1), and at the first visit after the combination therapy period (LAB2). Before finerenone introduction the patients received SGLT-2 inhibitors for 420 ± 300 (median 270) days. 

**Figure 1:**
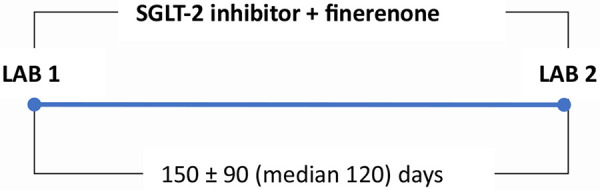
Duration of finerenone therapy period and laboratory timing. LAB1 (UACR1, UPCR1, **K**1, eGFR1) - before starting finerenone, LAB2 (UACR2, UPCR2, **K**2, eGFR2) - at the first visit after the combination therapy period. UACR = urinary albumin-to-creatinine ratio; UPCR = urinary protein-to-creatinine ratio; K = potassium; eGFR = estimated glomerular filtration rate.

The duration of therapy and timing of laboratory analysis during finerenone treatment are outlined in Figure 1. 

### Statistical analysis 

Descriptive statistics were calculated as the mean and standard deviation for normally distributed variables, and the median along with the interquartile range (IQR) for non-normally distributed variables. Normality was assessed using Kolmogorov-Smirnov test. Percentage changes in UACR, UPCR, and eGFR were calculated as the difference between values at the end and the beginning of a treatment period, divided by the value at the beginning of the period. Comparisons before and after finerenone were conducted using the paired-sample t-tests for normally distributed variables and the Wilcoxon signed-rank tests for non-normally distributed variables. A p-value of less than 0.05 was considered statistically significant. Data analysis was performed using IBM SPSS Statistics 25 (IBM Corp., Armonk, NY, USA). 

### Ethical approval 

The study was approved by the National Medical Ethics Committee of the Republic of Slovenia, Ministry of Health (approval number 0120-197/2025-2711-3). 

## Results 

The study cohort included 23 patients. Baseline demographic, clinical, and laboratory data before finerenone introduction are summarized in Table 1. 

Patients continued SGLT-2 inhibitor therapy throughout the combination therapy period, with 11 patients (48%) on empagliflozin (10 or 25 mg), 11 (48%) on dapagliflozin (10 mg), and 1 (4%) receiving both sequentially. Finerenone dosing remained stable during combination therapy, with 20 patients (87%) on 10 mg and 3 (13%) on 20 mg daily. Two patients were prescribed sodium zirconium cyclosilicate – 1 as chronic therapy, and 1 prophylactically due to a known predisposition to hyperkalemia, unrelated to finerenone. 

### Effects on albuminuria, proteinuria, and kidney function 

During combination therapy after finerenone introduction there was a trend toward a reduction in UACR, whereas UPCR decreased significantly. Additionally, there was a small decline in eGFR after finerenone introduction (Table 2). 

### Effects on serum potassium and other adverse events 

Serum potassium remained slightly elevated throughout the combination therapy period (Table 2). No clinically significant hyperkalemia was observed, although 1 patient had a mildly elevated potassium level of 5.6 mmol/L (above the threshold of 5.5 mmol/L) after finerenone initiation. No therapy adjustments were made. 

One patient (4%) reported occasional itching after starting finerenone, but this did not require discontinuation of treatment. 

## Discussion 

In our retrospective study, all patients received combination therapy with SGLT-2 inhibitors and finerenone. In addition to the expected benefits of SGLT-2 inhibitors, we observed a further reduction in albuminuria and proteinuria following the introduction of finerenone. This added effect was still evident despite patients having been on long-term treatment with RAS inhibitors and SGLT-2 inhibitors prior to finerenone initiation. The combination therapy was well tolerated and associated with only a slight, non-significant decline in kidney function. 

Real-world data on the effectiveness and safety of combined renoprotective therapies remain limited. In clinical practice, patients often receive combination therapies outside the strict criteria and predefined protocols of clinical trials. Observational studies, such as ours, better reflect real-world conditions where medication regimens vary and follow-up is tailored to individual needs. The global observational FINE-REAL study has already provided some interim results on finerenone treatment patterns and safety in real-world settings [16, 17]. The first randomized prospective CONFIDENCE study showed the combined benefits of finerenone and empagliflozin in diabetic kidney disease [18]. 

When comparing our findings to the global FINE-REAL study, both studies had a predominantly male cohort (74% in our study vs. 64.9% in FINE-REAL) and a high-risk CKD population (78% in FINE-REAL and a similar proportion in our study classified as high/very high risk by KDIGO). However, our cohort had a higher average age (72 vs. 66.5 years) and a longer duration of diabetes (median 20 vs. 14 years) compared to the second interim analysis of FINE-REAL [17]. 

Notably, the majority of our cohort received comprehensive renoprotective therapy: 91% were on RAS inhibitors, 100% on SGLT-2 inhibitors, and 100% on finerenone. In addition, 39% were also prescribed GLP-1 receptor agonists. This level of combined therapeutic coverage exceeds that reported in other studies [5, 14], including the real-world FINE-REAL study, where only 15% of participants received all three guideline-recommended therapies prior to starting finerenone [16, 17]. In our study, finerenone was prescribed by nephrologists based on laboratory results and in accordance with current recommendations [8]. Given the average eGFR of 45 ± 22 mL/min/1.73m^2^, most patients started with the recommended 10 mg dose. The patients continued the initially prescribed SGLT-2 inhibitor (empagliflozin or dapagliflozin) throughout the observation period. 

After finerenone initiation, we observed a small, non-significant decline in eGFR, a pattern that aligns with findings from previous studies, which reported similar early changes in kidney function without serious renal deterioration when combination therapies were used more extensively [13, 14]. This reduction in eGFR is likely the result of a synergistic hemodynamic effect of combination therapy, particularly in patients already receiving RAS and SGLT-2 inhibitors. However, this effect is generally considered non-pathological and is thought to reflect a mechanism that contributes to reduced albuminuria and long-term renoprotection. Over time, this translates into stabilization of kidney function and a slower progression of chronic kidney disease [4, 19]. During our observation period of approximately 5 months of combination therapy, it is possible that an initial pronounced decline in eGFR had already attenuated and consequently, after this extended follow-up, no significant early decrease in eGFR was detected. According to other studies, kidney function usually begins to stabilize between the 1^st^ and 3^rd^ month of combination therapy, even though there is an initial drop in eGFR due to hemodynamic changes [13, 18]. 

In our real-world study, finerenone was introduced at a moderate baseline UACR (280 mg/g (IQR 36 – 1,020)). Similarly, in the ongoing FINE-REAL study and in the finerenone randomized trials a baseline UACR was in the moderately to severely elevated range (median 300 and 830 mg/g, respectively) [5, 16, 17, 18]. Other observational studies on finerenone use in diabetic CKD have reported a successful reduction of even higher UACR (> 3,000 – 5,000 mg/g) [13]. The relatively lower baseline UACR in our cohort may reflect better regional lifestyle, more effective CKD prevention, and earlier nephrology referrals. 

Adding finerenone in our patients reduced the UACR by 36% and UPCR by 35%, with a significant decrease in UPCR, consistent with other observational studies comparing combination therapy to monotherapy [9]. In the recent randomized CONFIDENCE trial combination therapy with empagliflozin and finerenone reduced the UACR by 52% in 180 days, which was a bit longer period than in our study, with an ~ 30% greater reduction of UACR in combination therapy compared to either of the drugs alone. In combination therapy the full additive effect was shown as it was expected on the basis of the reductions observed with finerenone alone and empagliflozin alone [18]. One real-world observational study reported an even greater reduction in UACR of 73% after 180 days of combination therapy with SGLT-2 inhibitors and finerenone [13]. 

In our experience, sequentially introduced therapy was well tolerated. Of the 23 patients only 1 (4%) reported mild itching after starting finerenone, which did not require discontinuation of therapy. Itching and skin rash have also been reported as adverse effects of finerenone in another observational study [13]. The incidence of adverse reactions in our study was lower than in randomized and observational finerenone trials [5, 16], maybe due to shorter observational time and a small cohort of patients. 

Dual inhibition of renin-angiotensin-aldosterone system with RAS inhibitors and finerenone can increase the risk of hyperkalemia. Adding finerenone to RAS and SGLT-2 inhibitors in our study led to a significant increase in serum potassium. Despite this, no clinically significant hyperkalemia occurred, though 1 patient had a mild potassium elevation (~ 5.6 mmol/L) without requiring drug discontinuation. Similarly, a study combining finerenone with dapagliflozin reported a mild increase in potassium without serious hyperkalemia, consistent with our findings [9]. Nevertheless, combining finerenone with SGLT-2 inhibitors may lower the risk of hyperkalemia due to the potassium-excreting effect of SGLT-2 inhibitors; therefore, combination therapy is advised [10, 14, 20, 21]. 

Our study’s limitations include a small sample size and variable follow-up intervals with a relatively short combination therapy period with finerenone. These variations may affect the magnitude and timing of observed laboratory changes and adverse events. Additionally, some laboratory data were missing, which is common in real-world settings. 

In conclusion, even in this small cohort, we observed a trend toward reduced albuminuria and proteinuria, with relatively stable renal function when combination therapy with SGLT-2 inhibitors and finerenone, alongside RAS inhibitors and GLP-1 receptor agonists, was used. On the other hand, our study also provided a unique local perspective based on routine clinical practice, in contrast to global studies. 

Looking ahead, future research should aim to expand the cohort receiving sequentially introduced therapy and explore the effects of multiple therapy initiated simultaneously, as well as the impact of finerenone on non-diabetic patients. Larger observational studies with longer follow-up periods are needed to better understand the combination treatment in CKD patients. 

## Authors’ contributions 

M.A. conceived and designed the study; M.A., D.F., B.L., and A.A. helped with the inclusion of patients; M.A. and D.F. collected all the data, performed statistical analysis and wrote the manuscript. All authors read and approved the final version of the manuscript. 

## Funding 

None. 

## Conflict of interest 

None. 

**Table 1.** Baseline demographic, clinical, and laboratory (LAB1) characteristics of the study population, prior to finerenone introduction.

Gender, n (%)	17 (74) male, 6 (26) female
Age, years ± SD	72 ± 7
Duration of T2D, years ± SD	20 ± 10
Clinical diagnoses*, n (%)	Diabetic/hypertensive – 12 (52)
	Diabetic only – 7 (31)
	Diabetic with cardiorenal syndrome – 2 (9)
	Diabetic with IgA GN – 1 (4)
	Diabetic with sarcoidosis – 1 (4)
KDIGO CKD risk categories, n (%) **	Very high – 12 (52)
	High – 8 (35)
	Moderate – 3 (13)
RAS inhibitor use (ACEi or ARBs), n (%)	21 (91)
SGLT-2 inhibitor use, n (%)	23 (100)
GLP-1 RA use (including semaglutide), n (%)	9 (39)
Semaglutide only, n (%)	6 (26)

n = number of patients; T2D = type 2 diabetes; IgA GN = IgA glomerulonephritis; CKD = chronic kidney disease; RAS = renin angiotensin system; ACEi = angiotensin converting enzyme inhibitors; ARBs = angiotensin receptor blockers; GLP-1 RA = glucagon-like peptide-1 receptor agonists. *One patient underwent a kidney biopsy, which confirmed concurrent IgA glomerulonephritis; **CKD risk stratification according to KDIGO guidelines [8].

**Table 2.** Laboratory parameters before finerenone introduction when patients were on SGLT-2 inhibitors (LAB1) and after finerenone introduction with combination therapy use (LAB2).

Laboratory parameters	Before finerenone introduction	After combination SGLT-2 + finerenone	p-value	Relative (%) and absolute changes after combination treatment (median [IQR] or mean ± SD)
UACR (mg/g), median [IQR]	285 [36 – 1,020]	266 [57 – 1,006]	0.15	–36% (IQR: –65 to +14)
UPCR (g/g), median [IQR]	0.52 [0.18 – 1.35]	0.41 [0.16 – 1.78]	0.046	–35% (IQR: –53 to –8)
eGFR (ml/min/1.73m^2^), mean ± SD	45 ± 22	44 ± 21	0.13	–4% ± 13
Potassium (mmol/L), mean ± SD	4.3 ± 0.5	4.5 ± 0.4	0.045	+0.16 ± 0.37*

n = number of laboratory parameters; UACR = urinary albumin-to-creatinine ratio; UPCR = urinary protein-to-creatinine ratio; eGFR = estimated glomerular filtration rate. *Instead of relative change, absolute change (mmol/L) was calculated for potassium.
